# Computing cell state discriminates the aberrant hematopoiesis and activated microenvironment in Myelodysplastic syndrome (MDS) through a single cell genomic study

**DOI:** 10.1186/s12967-024-05496-x

**Published:** 2024-07-20

**Authors:** Xinyu Guo, Wenyan Jin, Yuchen Wen, Zhiqin Wang, Xiaotong Ren, Zhaoyun Liu, Rong Fu, Zhigang Cai, Lijuan Li

**Affiliations:** 1 Department of Hematology, Tianjin Medical University Tianjin General Hospital, Tianjin, China; 2 National Key Laboratory of Experimental Hematology, Tianjin, China; 3 Tianjin Key Laboratory of Inflammatory Biology, Tianjin, China; 4 The Province and Ministry Co-Sponsored Collaborative Innovation Center for Medical Epigenetics, Department of Pharmacology, School of Basic Medical Science, Tianjin Medical University, Tianjin, China; 5 Department of Rheumatology and Immunology, Tianjin Medical University Tianjin General Hospital, Tianjin, China; 6 Tianjin Key Laboratory of Bone Marrow Failure Malignant Hemopoietic Clone Control, Tianjin, China; 7 Tianjin Institute of Hematology, Tianjin, China

**Keywords:** Myelodysplastic syndrome, Single cell omics, Computational hematopoiesis, Bone marrow cell reference, HMGA1

## Abstract

**Background:**

Myelodysplastic syndrome (MDS) is a complicated hematopoietic malignancy characterized by bone marrow (BM) dysplasia with symptoms like anemia, neutropenia, or thrombocytopenia. MDS exhibits considerable heterogeneity in prognosis, with approximately 30% of patients progressing to acute myeloid leukemia (AML). Single cell RNA-sequencing (scRNA-seq) is a new and powerful technique to profile disease landscapes. However, the current available scRNA-seq datasets for MDS are only focused on CD34^+^ hematopoietic progenitor cells. We argue that using entire BM cell for MDS studies probably will be more informative for understanding the pathophysiology of MDS.

**Methods:**

Five MDS patients and four healthy donors were enrolled in the study. Unsorted cells from BM aspiration were collected for scRNA-seq analysis to profile overall alteration in hematopoiesis.

**Results:**

Standard scRNA-seq analysis of unsorted BM cells successfully profiles deficient hematopoiesis in all five MDS patients, with three classified as high-risk and two as low-risk. While no significant increase in mutation burden was observed, high-risk MDS patients exhibited T-cell activation and abnormal myelogenesis at the stages between hematopoietic stem and progenitor cells (HSPC) and granulocyte–macrophage progenitors (GMP). Transcriptional factor analysis on the aberrant myelogenesis suggests that the epigenetic regulator chromatin structural protein-encoding gene *HMGA1* is highly activated in the high-risk MDS group and moderately activated in the low-risk MDS group. Perturbation of *HMGA1* by CellOracle simulated deficient hematopoiesis in mouse Lineage-negative (Lin^-^) BM cells. Projecting MDS and AML cells on a BM cell reference by our newly developed MarcoPolo pipeline intuitively visualizes a connection for myeloid leukemia development and abnormalities of hematopoietic hierarchy, indicating that it is technically feasible to integrate all diseased bone marrow cells on a common reference map even when the size of the cohort reaches to 1,000 patients or more.

**Conclusion:**

Through scRNA-seq analysis on unsorted cells from BM aspiration samples of MDS patients, this study systematically profiled the development abnormalities in hematopoiesis, heterogeneity of risk, and T-cell microenvironment at the single cell level.

**Supplementary Information:**

The online version contains supplementary material available at 10.1186/s12967-024-05496-x.

## Introduction

MDS is a highly heterogeneous blood disorder and around 30% MDS patients will proceed to Acute Myeloid Leukemia (AML) [7, 22]. Except few MDS subtypes could be clearly defined by gene mutations (i.e. mutations in *SF3B1* induces the MDS-RS subtype), most of the MDS have no clear diagnosis criteria since they share numerous mutations in a panel of common genes and even have quite similar symptoms as other myeloid abnormalities (i.e. AML, chronic myelomonocytic leukemia CMML, or myeloproliferative neoplasm MPN), or autoinflammatory diseases (i.e. vacuoles E1 enzyme X-linked autoinflammatory somatic, VEXAS) [4, 9, 28, 36].

Single-cell-omics (SCO) studies are changing the field of clinical research including its practice and even the regular clinical management in future (i.e. diagnosis, drug responses, patient tailoring, and disease follow-up) [35]. In the past five years, numerous studies dissected the abnormalities of AML using unsorted or sorted BM cells for SCO analysis [1, 11, 30–32, 37, 40, 42]. With regards to the published SCO studies of MDS, most of them used sorted BM cells to profile hematopoietic alterations. To our knowledge, however, few studies so far directly profile entire BM cells of MDS patients. In the report by Guess et al., only two MDS patients scRNA-seq datasets were generated and their controls are the BM cell at their AML stage rather than the healthy donors [15]. Thus, most of the publicly accessible resources of scRNA datasets were analyzing only a small pool of BM biopsies, e.g. hematopoietic stem progenitor cells (HSPC), common myeloid progenitor cells (CMP) or granulocyte–macrophage-progenitor cells (GMP). To date, a comprehensive cell atlas of MDS and the differences or connections with other myeloid neoplasms are still lacking.

We are conducting a study to profile around 100 MDS patients using unsorted BM cells on single cell omics platforms. We argue that this cohort of unsorted BM cells will produce more valuable resource and a more comprehensive cell atlas for understanding MDS etiology, comparable to or even outperforming datasets of flow cytometry, especially when considering the continuous status of hematopoiesis in scRNA-seq analysis and the interaction between abnormal cells and their microenvironment [40, 41]. In this study, we report our preliminary results based on a mini-cohort of MDS patients (N = 5) along with healthy controls (N = 4). All of the original sequencing datasets were produced by a same single-cell RNA-sequencing platform (SeekOne, see Methods in detail) and the downstream analysis were conducted by rigorous filtering and computing.

## Materials and methods

### Patients

This study was conducted with the approval of the Tianjin Medical University General Hospital's Ethics Committee and participants were provided written informed consent prior to taking part in the study. The five treatment-naive MDS patients were enrolled in the study during June 2022 and June 2023 in the hospital**.** Clinical information of the enrolled MDS patients were summarized in Table 1. Of note, the patients MDS2 and MDS5 had somatic mutations in *DDX41* [33]; the patients MDS1 had somatic mutations in several genes including *SRSF2*, *RUNX1*, *PTPN11*.

**Table 1:** Clinical information of enrolled MDS patients

Patient Index	Gender	Age	Hematolo gical diagnosis	Other diagnosis	White blood cell (*10^9/L)	Hemog lobin (g/L)	Platelets (*10^9/L)	Expression of Leukemia- related Markers	Chromosomal karyotype	FISH	Detected mutations and the values of variant allele fraction (VAF)	Megakary ocyte	IPSSR
											BOOR (10.94%), DNMT3A		
											(exon19):		
											(11.4%);RUNX1(NM_00175.4:		
											EXON5:C.503G >		
								Partial expressesion of			T:p.Gly168Val): (14.42%);		
MDS1	Female	69	MDS-EB2	Psoriasis for ten years	1.08	86	137	CD34, HLA-DR, CD13, CD64, with weak expression	Normal	D20S108/CSP8[del (20q)] positive	RUNX1 (NM_00175.4:EXON5:C.497_	Normal	7.5
								of CD117, CD33			498insCA:p.Ser167fs):		
											(11.35%); SRSF2 (12.72%);		
											PTPN11 (12.63%);		
											DNMT3A(exon1): (50.33%);		
											WT1 gene negative		
MDS2	Male	67	MDS-EB2	Pneumonia	0.98	50	24	Positive expression of CD117, CD33, CD34 positive and partial expression of CD64 andCD13	Normal	Negative	ASXL: 11.06%; DDX41 (exon14): 32.96%; TP53: 2.38%	Very Low	8.5
								Abnormal cell population		CSF1R/D5S23,			
MDS3	Female	79	MDS-EB2	Acute myocardial infarction	2.43	66	18	detected with a fraction of 18.13%; Partial expression of CD117, CD33, CD11b, CD34, CD64, MPO, HLA-DR,	Normal	D5S721[del(5q33)] positive, EGR1/D5S23,D5S7 21[del(5q31)]positi	WT1, negative	Normal	8.5
								CD13		ve			
MDS4	Female	66	MDS-EB1	Pneumonia and liver dysfunction	1.16	53	22	Abnormal cell population detected with a fraction of 7.51%; Partial expression of CD117, CD33 and CD34	Normal	Negative	WT1, positive, CT = 29.93	Normal	5.5
								Abnormal cell population					
MDS5	Male	53	MDS-EB2	EB virus infection	1.3	129	67	detected with a fraction of 16.11%; partial expression of CD117, CD64, MPO, CD33,	Normal	Negative	DDX41 (exon15): 7.99%, DDX41 (exon14): 32.02%	Normal	7
								HLA-DR, CD13, CD34					

### Preparation of bone marrow cells

Fresh bone marrow blood was separate using Histopaque®-10771 (Sigma-Aldrich Catalog No.10771-6X100ML) as instructions. Cell count and viability was estimated using fluorescence Cell Analyzer (Countstar® Rigel S2) with AO/PI reagent after removal erythrocytes (Solarbio R1010). Finally, fresh cells were washed twice in the RPMI1640 and then resuspended at 1 × 10^6^ cells per ml in 1 × PBS and 0.04% bovine serum albumin.

### Single cell RNA-seq library construction and sequencing

Single-cell RNA-Seq libraries were prepared using SeekOne® Digital Droplet Single Cell 3’ library preparation kit (SeekGene Catalog No.K00202). Briefly, appropriate number of cells were mixed with reverse transcription reagent and then added to the sample well in SeekOne® chip. Subsequently Barcoded Hydrogel Beads (BHBs) and partitioning oil were dispensed into corresponding wells separately in chip. After emulsion droplet generation reverse transcription were performed at 42 ℃ for 90 min and inactivated at 80 ℃ for 15 min. Next, cDNA was purified from broken droplet and amplified in PCR reaction. The amplified cDNA product was then cleaned, fragmented, end repaired, A-tailed and ligated to sequencing adaptor. Finally, the indexed PCR were performed to amplified the DNA representing 3′ polyA part of expressing genes which also contained Cell Barcode and Unique Molecular Index. The indexed sequencing libraries were cleanup with SPRI beads, quantified by quantitative PCR (KAPA Biosystems KK4824) and then sequenced on illumina NovaSeq 6000 with PE150 read length.

### Cell clustering, annotation, and visualization by Seurat

The scRNA-seq dataset analysis was performed using Linux scripts and R language and the in-house computing platforms have been described [16, 24]. Standard preprocessing and quality controls were performed on the dataset using Seurat (version: 4.1). Dimension reduction and visualization of the datasets were achieved through UMAP (Uniform Manifold Approximation and Projection). To address batch effects, Harmony (version: 1.2.0) was utilized for dataset integration. Clusters were identified using Seurat's cluster-finder computation algorithm, while cell types were annotated based on expression of canonical tissue compartment markers.

### Pseudotime state of each cell and gene expression simulated by Palantir

After transferring Seurat object to the proper Anndata format, we then run the python package Palantir with default parameters [34]. Only HSPC, GMP, Pro_Neutrophil, Neutrophil and Monocytes were used for Palantir analysis for simplicity. We randomly selected an HSPC as the starting point for trajectory analysis. The pseudotime state and gene expression matrix of each cell per cell type or per group were extracted from the simulated datasets and then visualized by ggplot2.

### Analysis of transcriptional factors and their regulons by SCENIC

The SCENIC computation pipeline was executed using the Python package pySCENIC [2, 6], and Van [39]. Firstly, the log-normalized count matrix was utilized as input, along with a curated list of known transcription factors (TFs), to generate regulons based on their correlation with putative target genes. Secondly, by integrating the generated adjacency matrix with human cisTarget databases (10kbpUp10kbpDown and TSS ± 10 kbp), the regulons were refined through pruning targets that lacked enrichment for the corresponding TF motif. Lastly, cells were scored for each regulon with a measure of recovery of target genes from a given regulon.

### Single base substitution (SBS) mutations calling using scRNA-seq datasets

The biocomputational working platform SComatic is a newly developed toolkit designed for calling somatic mutations in cells, with any germline mutations being filtered out during the procedure [25]. Therefore, to our knowledge, SComatic is currently the most advanced algorithm available for measuring somatic mutations in MDS. The software SComatic was used to conduct SBS calling according to its tutorial (python scripts in Linux miniconda environment). Firstly, raw sequencing files (formatted as BAM and containing aligned sequencing reads for all cell types) were split into cell-type-specific BAMs using precomputed cell type annotations in Seurat. Secondly, base count information for each cell type and every position in the genome was recorded and merged into a single matrix as TSV files. Finally, variants were called and high-quality mutations were labeled as 'PASS' for downstream statistical analysis. Mutation burden (per sample) was normalized: dividing the number of mutations by the number of cells in each sample.

### Cell communication analysis

After standard analysis of the scRNA-seq datasets by Seurat, cell-to-cell communication analysis was conducted using R package CellChat (Version: 1.6.1) [17]. All cell types in BMs were included in the analysis. The comparison analysis was performed between the high-risk MDS group and healthy controls or between the low-risk MDS group and healthy controls.

### Construction of gene signatures and scoring bioactivities in each cell

We integrated classic gene sets to characterize different bioprocesses, generating gene lists for subsequent analyses. The function ‘AddMouduleScore’ in the Seurat package was employed to calculate the average expression levels for each cluster. All signatures were binned based on the average expression.

### Statistical analysis

Statistical analyses were performed using Prism 9 or R packages. If not stated elsewhere, differences of quantitative parameters were assessed using the* t*-test for data that was normally distributed, or nonparametric test for data that was not normally distributed. Wilcoxon test was performed to test for differences in Palantir pseudotime between conditions. Results with p < 0.05 were considered statistically significant. *, p < 0.05; **, p < 0.01; ***, p < 0.001; ****, p < 0.0001.

### The MarcoPolo pipeline and the gene-perturbation prediction by CellOracle

In brief, the MarcoPolo pipeline is a machine-learning based one-stop computing procedure for annotating bone morrow or blood cells and generating visualization results including the density map on the reference and cell-type-fraction data frames. Detailed computing protocol and pipeline for MarcoPolo will be described elsewhere by Ma and Cai (manuscript in preparation). For the gene-perturbation prediction, we first generated a high-quality Lineage-negative (Lin^−^) bone morrow cell rather than total bone marrow cells or LSK cells in-house from the WT mouse (cell number 7266), along with mutations in some critical genes or in some stressed condition (Wen and Cai, manuscript submitted). To reducing the computing burden, we filtered only 2000 cells with 30,665 genes for CellOracle analysis [19]. The developmental flow without perturbation was computed by Palantir as described above [34]. Perturbation score (the inner product) is calculated based on comparing the 2D vector and visualized by the dots on the grids. The positive inner product is shown in green (indicating developmental trajectory is strengthened or expedited) while the negative inner product is shown in purple (indicating developmental trajectory is reversed). Detailed protocol for gene-perturbation in our own mouse Lin^− ^BM datasets will be described elsewhere by Wen and Cai (manuscript submitted).

## Results

### Unsorted BM cells for single cell RNA-sequencing analysis

**Fig. 1:**
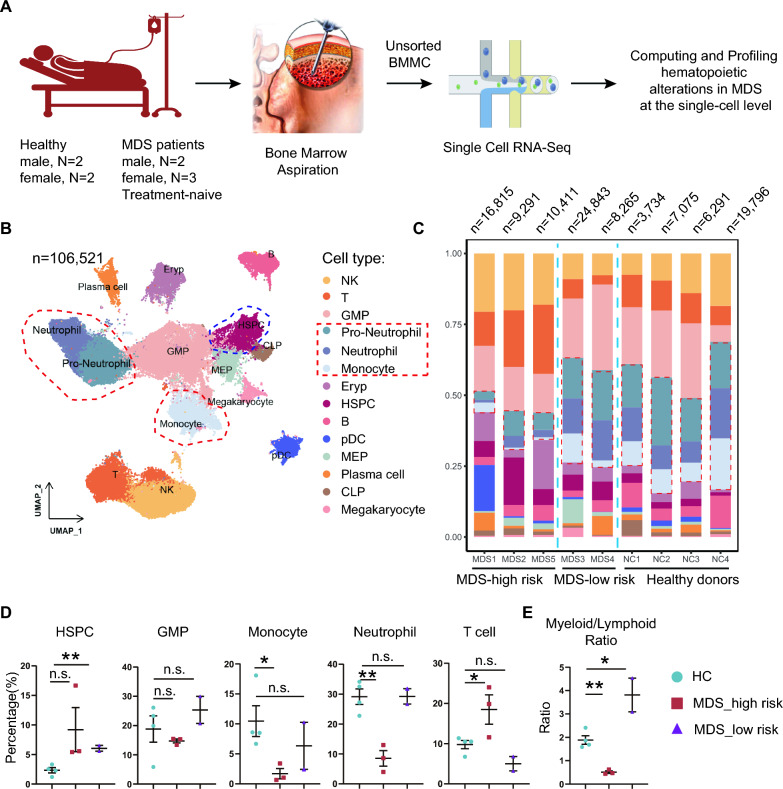
scRNA-Seq analysis of unsorted BM cells revealed hematopoietic abnormalities in the MDS patients. **A** Five MDS patients and four healthy controls donated the bone marrow biopsies for the single cell RNA-sequencing (scRNA-seq) study. See Table 1 for clinical information of the MDS patients. **B** In total 14 cell types were annotated in the UMPA plot. NK, nature killer cells; T, T cells; B, B cell; GMP, granulocyte–macrophage progenitor cells; MEP, megakaryocyte-erythroid progenitors; Eryp, erythroid progenitors; pDC, plasmatic dendric cell. **C** Bar plot for fraction of each cell type in the samples. The cell numbers of each sample are shown on the top of the panel. Notice that the high-risk MDS group has a much less fraction of pro-neutrophils, neutrophils and monocytes. **D** Quantitation of fractions of indicated cell types in the unsorted bone marrow cells. **E** The ratio of myeloid/lymphoid cells. n.s. not significant; *, p < 0.05; **, p < 0.01

The five treatment-naive MDS patients and four health donors were enrolled in the study during June 2022 and June 2023 in the hospital (Fig. 1A). The clinical information of the 5 MDS patients is briefed in Table 1. Of note, the MDS patients were detected with chromosomal normal and most of them were detected with mutations in clonal hematopoiesis-driver genes such as *DNMT3A*, *TP53*, *ASXL1* and *WT1* (with very low to very high variant allele fraction [VAF] value). When they were hospitalized, one was reported with 10-year psoriasis (Patient Index: MDS1), one was with EB virus infection (Patient Index: MDS5), one with acute myocardial infarction (Patient Index: MDS3) and two with pneumonia (Patient Index: MDS2 and MDS4).

After standard pre-processing, a total of 106,521 cells were filtered for downstream analysis (69,625 cells from 5 MDS patients and 36,896 cells from 4 healthy donors, respectively; range: 3734–24,843 cells per sample). In total 14 cell types were meticulously annotated in the Uniform Manifold Approximation and Projection (UMAP) presentation (Fig. 1B). In line with the clinical diagnosis, we clearly observed 3 out of 5 MDS patients have dramatic low production of both pro-neutrophils, neutrophils, and monocytes (Fig. 1C). Another two MDS patients have comparable fraction of these mature myeloid cells but higher HSPC fractions in their BM (Fig. 1C). Consistent with the 10-year psoriasis history (Fig. 1C, Patient Index: MDS1), a large fraction of plasmatic dendric cells (pDC) was identified in the UMAP plot, which is also revealed by flow cytometry (data not shown).

To facilitate the downstream analysis, we classified the 3 MDS patients with extreme low level of mature myeloid cells as the high-risk MDS group and the other two as the low-risk MDS group. Quantification of each cell type suggests that both MDS groups have greater fraction of HSPCs (Fig. 1D). The fractions of GMP in the three groups appear to be comparable. However, the fractions of neutrophils and monocytes are significantly decreased in the MDS-high risk group, indicating deficient myelogenesis in patients MDS1, 2 and 5 (Fig. 1E). The ratio of myeloid/lymphoid appears to be opposite between high-risk MDS and low-risk MDS, suggesting a transition stage probably exists between the groups in this blood disorder.

### Superfast annotation of BM cells by MarcoPolo pipeline reveals a connection between MDS and AML

**Fig. 2:**
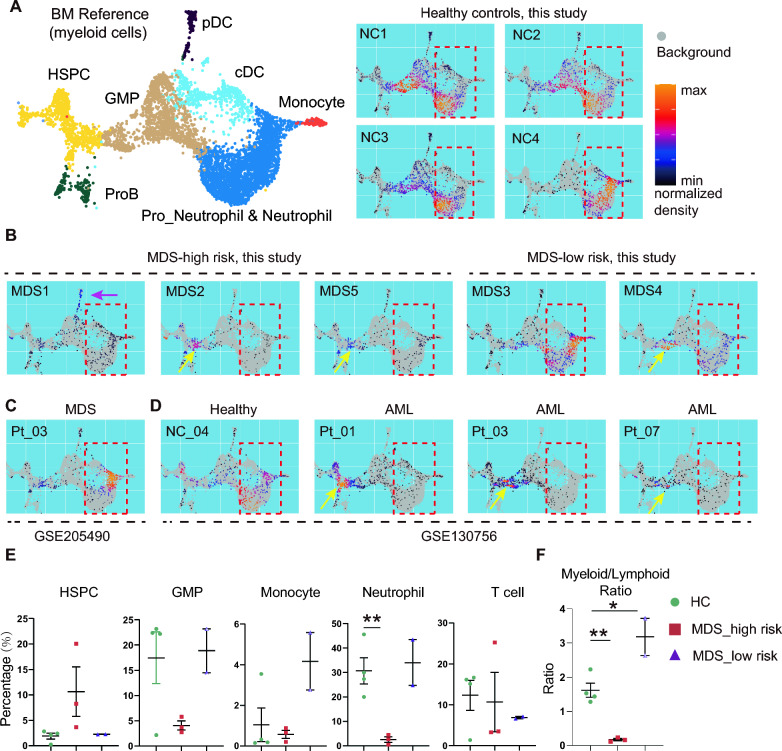
Projection of unsorted BM cells on the BM cell reference describes the connection between MDS and AML. **A** Density plot of 4 healthy donors on the BM cell reference map using MarcoPolo working flow (See the text and Methods for details; The cropped reference map shows 8 major cell types as indicated (left panel). A balanced density plot is visualized for each of the four healthy donors (right panel). Notice high density of cells in the highlighted region (dashed line). **B** Density plot of 5 MDS patients. 4 out of 5 MDS demonstrate very low cells in the highlighted region. High density of pDC in MDS1 is also noticed (pink arrow). Please also notice the abnormal high density of GMPs in the MDS2, 5 and 4 (yellow arrows). **C** Density plot of an MDS patient from the study GSE205490. **D** Density plot of a healthy donor and 3 AML patients from the study GSE130756. **E** Quantitation of fractions of indicated cell types in the unsorted bone marrow cells based on the values generated by the MarcoPolo pipeline. **F** The ratio of myeloid/lymphoid cells based on the values generated by MarcoPolo pipeline.*, p < 0.05; **, p < 0.01

To test if an automatic identification of BM cells facilitates our long-term clinical studies, we developed a working pipeline named as MarcoPolo which is able to reliably annotate ten thousand cells (a regular size of a scRNA-seq dataset for a BM sample) and visualize the dataset in an intuitive density map in 3 min (Ma and Cai, manuscript in preparation). In brief, we first generate a reference map of BM cells covering both progenitor cells and mature cells defined by gene expression matrix. The reference of myeloid linage cells is shown in Fig. 2A. When the datasets from the 5 MDS patients and 4 healthy donors were analyzed by MarcoPolo pipeline, the results are comparable to the meticulous annotation as shown in Fig. 1. We notice that the 4 healthy donors have balanced production of HSPCs, GMPs, and mature myeloid cells (monocytes and neutrophils) (Fig. 2A). However, the 3 MDS patients from the high-risk MDS group show much low density of mature myeloid cells (Fig. 2B). We also validate the MarcoPolo pipeline using MDS BM cells from the study (GSE205490), and healthy and AML BM cells from the study (GSE130756) [15, 42]. Consistently, the MacoPolo working flow efficiently and intuitively discriminates AML from healthy in 3 min per sample with ~ 10,000 cells (Fig. 2C and D). The quantification of BM cells by MarcoPolo pipeline is also comparable to our meticulous annotation (Fig. 2E and F), compared with Fig. 1D and E). This result suggests the MarcoPolo pipeline is able to quickly and accurately annotate BM cells including leukemic cells in a larger scale of cell numbers or in a larger cohort of enrolled samples.

### Computing the hematopoietic trajectory demonstrates an aberrant granulocyte genesis in the high-risk MDS group

**Fig. 3:**
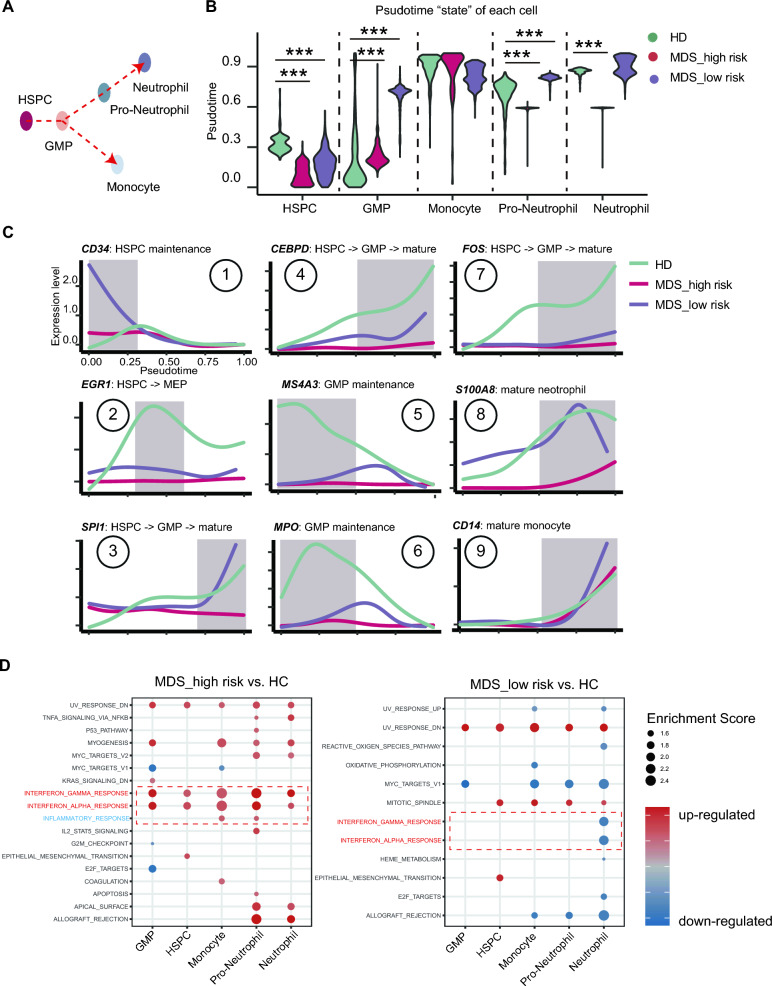
Aberrant and deficient hematopoiesis visualized by simulating the developmental time of each cell. **A** A schematic illustrating the developmental trajectory from HSPCs to neutrophils and monocytes. **B** Violin plot of the indicated cell types. The pseudotime value of each cell is simulated by the algorithm Palantir. **C** Expression of indicated genes along the pseudotime axis. The function of each gene is also labeled according to the experimental results. **D** The high-risk MDS group manifests overactivated interferon alpha and gamma signaling along with increased inflammation response while the low-risk MDS group does not. ***, p < 0.001

The developmental trajectory from HSPC to mature neutrophils and monocytes has been well studied experimentally using human or mouse BM cells [26, 29]. The development route is illustrated in Fig. 3A. When simulating the pseudotime of the MDS cells using the diffusion map-based algorithm Palantir (see related literatures and Methods for detail) [34], we observed that the pseudotime state of the HSPCs and GMP cells were significantly altered in both high-risk and low-risk MDS groups (Fig. 3B). Monocyte development appears normal. However, a procrastinated state was observed during pro-neutrophil and neutrophil development, which is consistent with decreased productions of these two cell types in the high-risk MDS group (Fig. 3B).

We also analyzed the expression of well-established markers or transcriptional factors in hematopoiesis. The difference in their expression is consistent with our expectation based on the ground-truth experimental results. For example, the expression of HSPC marker *CD34* is overactivated in the two MDS groups (Fig. 3C#1) while the expression of GMP maintenance markers *MS4A3* and *MPO* are repressed (Fig. 3C#5, #6). Accordingly, expression of mature neutrophil markers including *SPI-1*, *CEBPD*, *FOS, S100A8* are also decreased (Fig. 3C#3, #4, #7, #8). Expression of mature monocyte marker *CD14* appears to be normal (Fig. 3C#9). Interestingly, the expression of megakaryocyte-erythroid progenitor (MEP) marker *EGR1* appears to be repressed in MDS group (Fig. 3C#2), which is consistent with that anemia and thrombocytopenia are generally found in MDS patients. When analyzing altered pathways, we also found the high-risk MDS group has a typical upregulation of Interferon alpha and gamma signaling and inflammatory pathways, which is not observed in the low-risk MDS group (Fig. 3D). Taken together, these results demonstrate that the development abnormalities in the high-risk MDS group is faithfully detected by the pseudotime simulations using the scRNA-seq datasets.

### The chromatin epigenetic regulator HMGA1 and its regulons are overactivated in the high-risk MDS patients

**Fig. 4:**
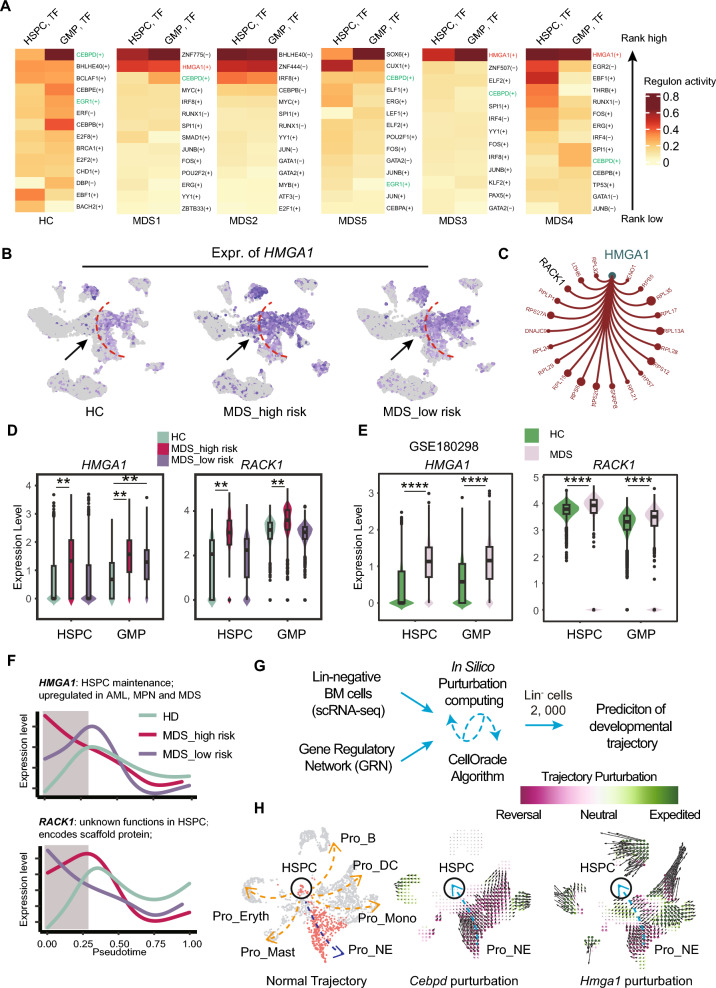
HMGA1 encodes a critical transcription factor for hematopoiesis but is overactivated in the high-risk MDS group. **A** The heatmap plot of top 14 transcriptional factors according to their regulon activity in HSPCs and GMP cells. Notice that in 4 out of the 5 MDS patients, only 1–3 transcriptional factor regulons are dominated the cells while in the healthy donors, several transcriptional factors are balanced well. **B** Expression of *HMGA1* on the UMAP plots. **C** RACK1 is predicted as one of effector downstream genes regulated by *HMGA1*. **D** Quantification of the gene expression level of *HMGA1* and *RACK1* in the indicated groups. **E** Quantification of the gene expression level of *HMGA1* and *RACK1* in the published study GSE180298. **F** Expression of *HMGA1* and *RACK1* along the pseudotime axis. The function of *HMGA1* is probably involved in regulating maintenance of HSPCs while that of *RACK1* in hematopoiesis is largely unknown. **G** A recently developed algorithm CellOracle is able to predict cell trajectory after perturbating certain transcriptional factors. See Methods for details. **H** We generated a high-quality dataset of mouse Lin^-^ bone marrow cells for predicting the consequence of *Cebpd* and *Hmga1*. As shown in the middle and right panel, the perturbation of *Cebpd* and *Hmga1* results in development deficiency of neutrophil production but increased production level of HSPC and GMP. Perturbation score (the inner product) is calculated based on comparing the vectors and visualized by the dots on the grid. The positive inner product is shown in green (indicating developmental trajectory is strengthened and expedited) while the negative inner product is shown in red (indicating developmental trajectory is reversed) **, p < 0.01; ****, p < 0.0001

To identify critical transcriptional or epigenetic regulators in the MDS single cell transcriptomic datasets, we then performed unbiased data-mining on the transcriptional factors and their regulons (downstream effector genes) using the algorithm SCENIC, SCENIC-plus [2, 6], and Van [39]. We first rank the regulon activities in HSPCs and GMP cells. The activity heatmap of top 14 regulons are shown in Fig. 4A. We notice that the regulon activities are gradually distributed in the healthy controls while 4 out of 5 MDS patients were dominated by 1 to 3 regulons. For example, in the HSPCs and GMP cells from the MDS1, 3, 4 patients, the *HMGA1* regulon dominated the cell pools. The rank level of *HMGA1* regulon is also elevated while that of *CEBPD* and *EGR1* were decreased. *HMGA1* has been reported to be overactivated in AML, MPN and MDS [23]. Here we then examined its expression level using the single cell datasets. As shown in Fig. 4B and D), expression of *HMGA1* is uniformly and significantly overactivated in the MDS-high risk group. Accordingly, we also validated its overactivation in the published MDS scRNA-seq dataset using only CD34^+^ BM cells (GSE180298, Fig. 4E). As the scaffold-encoding gene *RACK1* is predicted as a downstream effector of *HMGA1*, we validated that its expression is also upregulated in high-risk MDS group (Fig. 4C and D).

To further validate the results from the above analysis, we used scRNA-seq datasets of sorted CD34^+^ progenitor cells from patients with del(5q) MDS (GSE245452) (https://www.ncbi.nlm.nih.gov/geo/query/acc.cgi?acc=GSE245452), we found that expression of *HMGA1* and *RACK1* was also significantly elevated in the MDS group. Meanwhile, using bulk RNA-seq datasets of CD34^+^ progenitor cells from 159 patients with MDS patients and 17 healthy controls (GSE58831) [14], we found that expression of *HMGA1* was significantly elevated in the MDS group (Supplementary Fig. 1A–B). We simulated the expression of *HMGA1* and *RACK1* in the pseudotime axis and the results indicate its role is probably critical for HSPC and GMP maintenance (Fig. 4F). Meanwhile, it has been reported that the reduction of *HMGB1* is sufficient to impair HSC self-renewal and promote apoptotic cell death in MDS and that inhibitors of *HMGB1* signaling provided a first-in-class therapeutic option for patients with MDS[44]. Our results also confirmed the high expression of *HMGB1* in HSPCs and GMP cells of MDS (Supplementary Fig. 1C–E). Taken together, these results suggest that HMGA1 and HMGB1 both are likely involved in pathogenesis of MDS.

Furthermore, we generated a mouse wildtype Lin^−^ BM cells with high quality for CellOracle analysis (Fig. 4G; See Methods for detail) [19]. In the mouse Lin^−^ UMAP plot, 6 branches were annotated (Fig. 4H). Upon perturbation of *Cebpd* and *Hmga1*, we successfully simulated aberrant hematopoiesis (higher fraction of HSPCs and low fraction of committed progenitor cells), reminiscent of MDS phenotypes in human (Fig. 4H). Taken together, our results suggest that *HMGA1* and its regulon are over activated in the high-risk MDS group. Our study warrants that reducing the expression or activity of *HMGA1* represents an important strategic option for ameliorating MDS in future study.

### T cell microenvironment is actively mobilized in the high-risk MDS

**Fig. 5:**
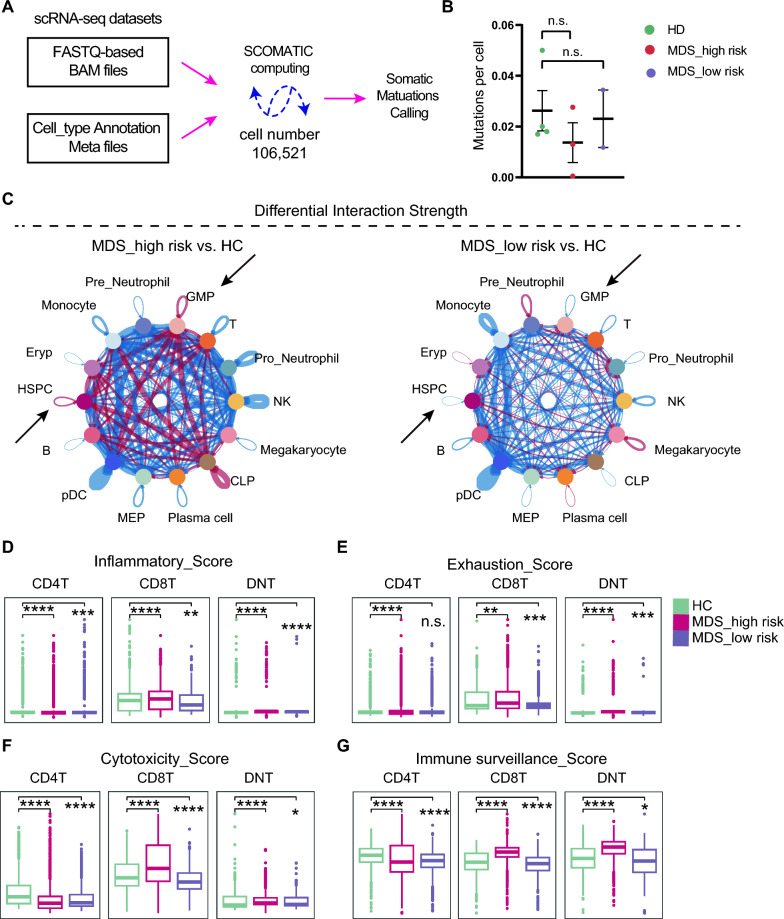
T cell microenvironment is actively mobilized in the high-risk MDS. **A** Somatic mutation calling was conducted by SComatic computing. See Methods for details. **B** No significant difference of mutation burden was found in the three groups. **C** Circular plots showing the overall differential cell–cell communications strength between high-risk MDS group and healthy controls or between low-risk MDS group and healthy controls. Red lines indicate an increased interaction while blue lines indicate a decreased interaction. **D–F** Scoring T cell activity based the gene sets related to T cell inflammation, T cell exhaustion, T cell cytotoxicity and T cell immune surveillance. *, p < 0.05; **, p < 0.01; ***, p < 0.001; ****, p < 0.0001

During the diagnosis, we notice several clonal hematopoiesis-related gene were mutated (see the Table 1). We ask if increased somatic mutations exist in MDS patients compared with the healthy controls. We performed the somatic mutation calling at the coding-sequence wide by the SComatic pipeline using the scRNA-seq datasets (Fig. 5A; See Methods for detail) [25]. As shown in Fig. 5B, no significant somatic mutation burdens were observed. We then ask if cell-to-cell chat is altered in the MDS patients. As shown in Fig. 5C, the high-risk MDS group manifests much greater strength of cellular communication in the BM environment, especially when HSPCs and GMPs were calculated. When scoring the activity of T cells in the MDS groups, we also observed that high-risk MDS group have increased inflammation level, exhaustion activity, cytotoxicity activity and immune surveillance level for CD8^+^T and DNT cells (Fig. 5D–G). Based on these side-by-side comparisons using the T cells form the scRNA-seq datasets, we concluded that T cells are more activated in the high-risk MDS group than that in the low-risk MDS group.

## Discussion

In the study we report an interim result of our long-term 100 MDS scRNA-seq project. Integration of the large BM datasets using a regular algorithm or by the MarcoPolo pipeline warrant that we can handle million BM cells in future. The MarcoPolo pipeline is able to quickly and accurately annotate BM cells including leukemic cells in a larger scale of cell numbers or in a larger cohort of enrolled samples. The open-access availability of the large cohort of BM datasets will assist our understanding the pathology and heterogenicity of MDS.

Distinct from previous single cell omic studies using sorted BM cells [3, 5, 8, 13, 15, 21, 38, 43], here we aim to profile all of the BM cells from healthy donors or patients with MDS (as described in this study) and other blood disorders (our other long-term goals). Even in the mini-cohort of MDS in the study, we observed heterogenicity of this complicated blood disorders. Based on the frequencies of mature myeloid cells, we stratified the 5 MDS patients into two groups: high-risk and low-risk group. Interestingly, both groups manifest a higher proportion of hematopoietic stem and progenitor cells (HSPCs). The results indicate that bone marrow dysplasia in MDS patients display malignancies at different grades or at different developmental stages. This may be related to the selective growth advantage of somatically mutated clonal HSPCs in the hematopoietic system [27]. The proportions of neutrophils and monocytes exhibit a significant decrease in the MDS-high risk group, suggesting impaired myelogenesis in patients MDS1, 2, and 5. The pathway enrichment results show that the high-risk group of MDS patients is enriched in the upregulation of interferon alpha and gamma signaling pathways, while the low-risk group of MDS patients is enriched in the UV response and epithelial-mesenchymal transition pathways. This observation is in accordance with previous reports demonstrating increased inflammatory signaling in the disease [12, 20].

Our study also shed light into the molecular pathogenesis of MDS at the transcriptional or epigenetic regulation level. Transcription factor analysis results show that the regulators of each cell type differ between MDS patients and healthy donors. Each MDS patients showed alterations of specific regulons, indicating that the heterogeneity of the disease, which is not possible to be observed by regular flow cytometry. When comparing the healthy control and MDS group, we also notice that the ranks of regulons in HC appear to be diversified while that in MDS is dominated by one or two regulons. Importantly, we prioritized that *HMGA1* should be appreciated in the future MDS studies. *HMGA1* is involved in various cellular processes such as transcriptional regulation, DNA repair, cell differentiation and regulated cell death. In our MDS patients (3 out of 5), the activity of HMGA1 regulon is significantly higher than in the healthy group. When comparing its activity within the MDS group, we also noticed that *HMGA1* expression level in the high-risk group of MDS is significantly higher than that in the low-risk group. We also validated the result using additional public datasets. This observation is in accordance with previous reports demonstrating distinctly upregulated of *HMGA1* in these MDS [10] and AML patients [23]. We think the upregulation of *HMGA1* contributes to the pathogenesis of MDS likely through blocking normal myeloid differentiation. *HMGA1* gene may play a key role in controlling the occurrence and progression of MDS patients. The risk degree of MDS patients could be assessed based on the expression level of the *HMGA1* gene.

In addition, we also found that one of the HMG family memers *HMGB1* is highly expressed in MDS datasets from GSE245452 and GSE180298. Kam et al*.* discovered that High Mobility Group Box-1 (HMGB1) had increased expression in primary CD34^+^ MDS cells compared to healthy CD34 + hematopoietic cells [18]. It has been suggested that *HMGB1* may be a very useful biomarker for the diagnosis and prognosis of hematological malignancies [44]. Our results are consistent with these studies, indicating that *HMGB1* is also a therapeutic target in MDS. In addition, our study found SMAD1 regulon was active in patient MDS1, showing its highest activity in HSC and GMP cell types. Patient MDS1 presented an ubiquitous activity of the regulon guided by POU2F2, a transcriptional factor overexpressed in AML. *SMAD1* and *POU2F2* has been shown to be highly active in patients with MDS [3].

Finally, in addition to interferon signaling mentioned above, we also observed changes in the immune microenvironment of MDS patients. We showed that T cells exhibit a higher level of activation in the high-risk MDS group. Single-cell omic datasets with genetic mutation profiles and gene expression will further assist understanding the interaction of malignant and non-malignant cells. Nonetheless, our study certainly has several limitations since the mutant or malignant cells in the BM scRNA-seq dataset are not well defined in the present study. Including the mutation information, malignant information, and even lineage/phylogeny information in the datasets will greatly assist us stratify different BM cells in the MDS pathogenesis.

In the future, if feasible, larger cohorts for comparing MDS with other blood disorders including myeloid leukemia, VEXAS, aplastic anemia are desired. Extending the cohort to 50 MDS patients or 100 MDS patients from multiple centers, or longitudinal collecting MDS BM samples with drug treatments will also assist us to have better understanding the development of this complicated blood disorders.

## Conclusions

As an initial outcome of our on-going large cohort of MDS single cell omics project, we successfully profiled deficient hematopoiesis in the high-risk MDS group using unsorted bone marrow cells in the study. We developed several computing tools or working pipelines to determine the alterations in MDS and its relationship with AML or other blood disorders. Importantly, our study warrants that chromatin-dynamics-related epigenetic regulator *HMGA1* is reprogrammed during hematopoiesis in MDS. Targeting *HMGA1* should be appreciated for interfere MDS development. In summary, the present study warrants that single cell omics studies generate valuable and comprehensive cell atlas for understanding blood disorders including MDS.

### Supplementary Information

Additional file 1.

## Data Availability

The scRNA-seq datasets of this study will be accessible to the public upon the publication. The validation datasets for BM cells from healthy, AML and MDS donors were downloaded from the sources. GSE130756, GSE180298, GSE205490 were described in the reports [3, 15, 42].

## Abbreviations

MDS: Myelodysplastic syndromes
BM: Bone marrow
scRNA-seq: Single-cell RNA sequencing
AML: Acute Myeloid Leukemia
CMML: Chronic myelomonocytic leukemia
MPN: Myeloproliferative neoplasm
SCO: Single-cell-omics
HSPC: Hematopoietic stem progenitor cell
CMP: Common myeloid progenitor
GMP: Granulocyte–macrophage-progenitor
VEXAS: Vacuoles E1 enzyme X-linked autoinflammatory somatic
MEP: Megakaryocyte-erythroid progenitor
