# Supplementary figures and images for: Computing cell state discriminates the aberrant hematopoiesis and activated microenvironment in Myelodysplastic syndrome (MDS) through a single cell genomic study
Source: J Transl Med. 2024 Jul 20;22:673. doi: 10.1186/s12967-024-05496-x (PMC11265062; doi:10.1186/s12967-024-05496-x)

**A**

GSE245452

HC  
MDS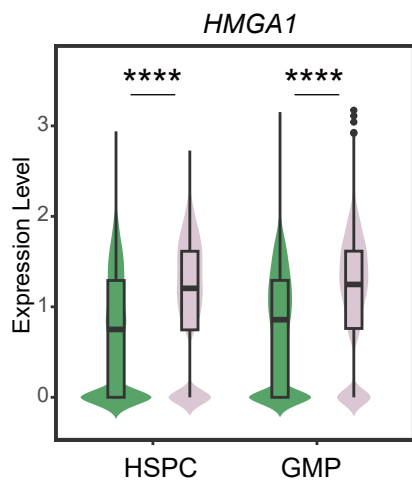**B**

GSE58831

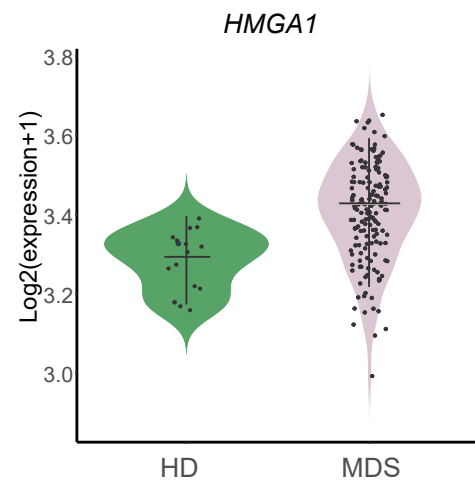**C**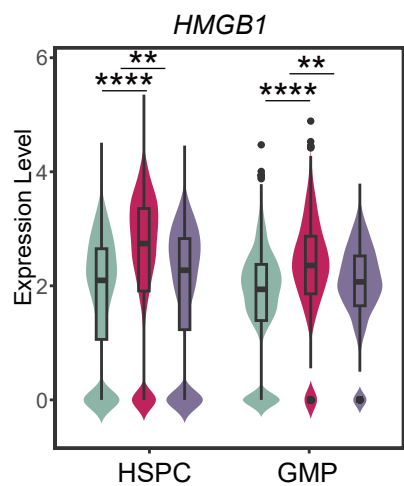**D**

GSE245452

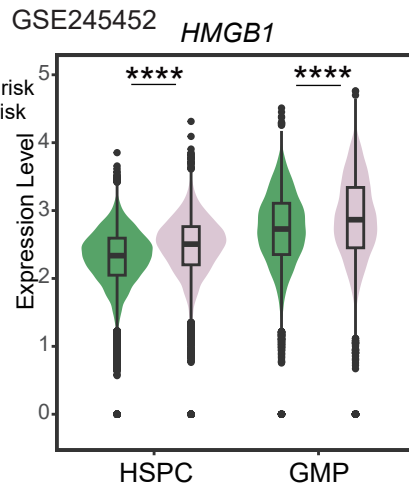**E**GSE180298 *HMGB1*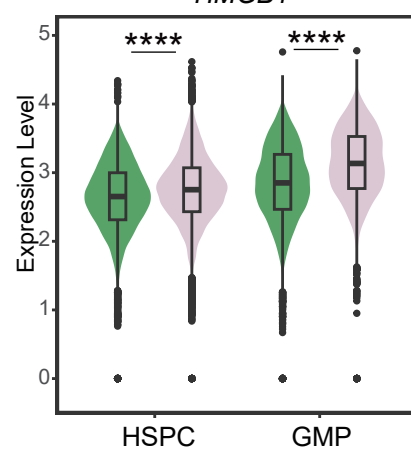

Supplement: Supplementary file 1 — Additional file 1. [file 12967_2024_5496_MOESM1_ESM.pdf]
